# Predicting One-Year Psychiatric Rehospitalization Using Natural Language Processing and Machine Learning Methods Based on Clinical Texts

**DOI:** 10.3390/jcm15197457

**Published:** 2026-09-25

**Authors:** Sare Aydın, Sena Karaca, Esma Akpınar Aslan, İlker Güneysu, Figen Ünal Demir, Filiz Özsoy, Burcu Eser

**Affiliations:** 1 Department of Psychiatry, School of Medicine, Tokat Gaziosmanpaşa University, 60250 Tokat, Türkiye; 2 Department of Psychiatry, Tokat State Hospital, 60100 Tokat, Türkiye

**Keywords:** psychiatric readmission, length of hospital stay, natural language processing

## Abstract

**Background/Objectives:** Psychiatric readmissions are an important indicator for assessing disease progression and the effectiveness of healthcare services. The aim of this study was to explore factors associated with one-year readmission and to evaluate the performance of machine learning models using clinical information extracted from discharge summaries of patients receiving inpatient treatment in a psychiatric ward. **Methods:** This retrospective study included 622 patients hospitalized in the psychiatry department of a university hospital between 2020 and 2024. Clinical variables were extracted from discharge summaries using a theme-based structured coding approach. Factors associated with 1-year readmission were evaluated using univariate and multivariable logistic regression analyses, while length of hospital stay was analyzed using log-transformed linear regression. The predictive performance of logistic regression, Random Forest, HistGradientBoosting, and BERTurk-based machine learning models was also compared. **Results:** The 1-year psychiatric readmission rate was 17.0%. In univariate analyses, previous psychiatric hospitalization, longer index length of stay, and manic symptoms were associated with readmission. In multivariable analysis, only previous psychiatric hospitalization remained an independent predictor of readmission (adjusted OR = 2.14, 95% CI: 1.34–3.42; *p* = 0.001). Previous psychiatric hospitalization, unemployment, and depressive symptoms were independently associated with longer hospital stay. Among the machine learning models, logistic regression using structured clinical variables and theme-based features achieved the best performance (AUC = 0.602). **Conclusions:** Previous psychiatric hospitalization was the strongest independent factor associated with 1-year psychiatric readmission. These findings suggest that routinely collected clinical records may contain potentially useful information for understanding psychiatric readmission risk and support further investigation in larger, multicenter studies.

## 1. Introduction

Mental disorders are characterized by impaired functioning and reduced quality of life, and many follow a chronic course marked by recurrent episodes and periods of remission. Consequently, repeated psychiatric hospitalizations are common across many mental disorders and represent a significant clinical challenge. A substantial proportion of psychiatric admissions are followed by readmission within a short period after discharge. Previous studies have reported unplanned psychiatric readmission rates of up to 43%, exceeding those observed following general medical hospitalizations [1,2,3]. Recurrent hospitalizations are associated with further functional decline, reduced quality of life, and a considerable economic burden on healthcare systems [4,5]. Therefore, identifying patients at increased risk of readmission is of considerable clinical importance for optimizing discharge planning and guiding post-discharge outpatient follow-up.

Systematic reviews examining predictors of psychiatric readmission have consistently identified previous psychiatric hospitalization as the strongest and most reliable predictor among the many variables investigated [6,7,8]. Patients with a history of recurrent hospitalizations have been shown to face a progressively increasing risk of future admissions, with 1-year readmission rates approaching 50% in some cohorts [9]. In addition, systematic reviews and observational studies have identified psychotic disorders, lower educational attainment, unemployment, single marital status, socioeconomic deprivation, and a history of involuntary hospitalization as important risk factors for psychiatric readmission [1,10,11,12]. These findings suggest that psychiatric readmission is a multidimensional outcome influenced not only by clinical characteristics but also by patients’ socioeconomic circumstances and patterns of previous healthcare utilization. In contrast, the associations of variables such as length of hospital stay and sex with readmission have been inconsistent across studies [12].

In addition to recurrent psychiatric readmissions, factors influencing length of hospital stay have received increasing attention in recent years. Current evidence suggests that hospital stay is determined by a complex interplay of patient-, disease-, and healthcare system-related factors. Psychotic disorders, greater illness severity, a history of previous psychiatric hospitalization, prior suicide attempts, and lower socioeconomic status have been associated with longer hospital stays, whereas younger age and voluntary admission have been linked to shorter stays [13,14]. However, the determinants of length of stay may vary according to the organization of healthcare systems, the availability of mental health services, and country-specific socioeconomic conditions, highlighting the importance of generating context-specific evidence [15].

With the increasing availability of health data, text analytics and natural language processing (NLP) have become increasingly important for extracting clinically relevant information from unstructured textual data. Healthcare records contain substantial amounts of valuable information in narrative forms, including diagnostic records and discharge summaries. Health NLP, as an interdisciplinary field combining natural language processing and healthcare, supports the systematic processing, extraction, and interpretation of information from clinical narratives and has been increasingly applied across a broad range of healthcare applications [16,17].

With the widespread adoption of electronic health records, approaches for predicting psychiatric readmission have become increasingly diverse [18,19]. In addition to structured clinical data, information extracted from free-text clinical documents, such as discharge summaries, using natural language processing (NLP) has been shown to improve predictive models. However, the generalizability of text-based models across different hospital information systems and clinical settings remains limited, and models developed at a single center often demonstrate reduced performance in external validation studies [19]. Accordingly, interest in applying machine learning methods to predict psychiatric readmission and other clinical outcomes has grown substantially in recent years. Unlike traditional statistical approaches, machine learning algorithms can model complex, nonlinear relationships among large numbers of variables, offering important advantages for individualized risk prediction [20].

The primary aim of this study was to examine the associations of demographic, clinical, and text-derived factors for 1-year psychiatric readmission using electronic health records and discharge summaries of patients hospitalized in a psychiatric inpatient unit. As a secondary analysis, we examined demographic, clinical, and text-derived factors associated with index hospitalization length of stay.

## 2. Materials and Methods

Electronic health records from the psychiatry department of a university hospital were screened for the period between 1 January 2020 and 31 December 2025. Patients hospitalized between 1 January 2020 and 31 December 2024 were included in the study. The dataset comprised 1064 hospitalization records from 751 unique patients. For each patient, the first hospitalization during the study period (2020–2024) was defined as the index hospitalization. Patients whose first hospitalization occurred in 2025 (*n* = 129) and who had no admissions during the index period were excluded. The final study cohort consisted of 622 patients. The primary outcome was psychiatric readmission to the same hospital within 365 days after the index hospitalization. The primary analysis focused on exploring factors associated with 1-year psychiatric readmission and evaluating model discrimination.

Because the data were anonymized prior to analysis, admission dates were represented at the calendar-quarter level rather than as exact dates. Accordingly, 1-year psychiatric readmission was defined using the calendar-quarter difference between the index hospitalization and the subsequent hospitalization. Readmissions occurring within the same quarter or up to three quarters after the index hospitalization were classified as occurring within 1 year, as these intervals are necessarily ≤365 days. A four-quarter interval was considered indeterminate because it may or may not fall within 365 days, and these cases were excluded from the primary 1-year readmission outcome. Intervals of five or more quarters were considered to exceed 1 year. Thus, the quarter-based definition represents a proxy for the true 365-day readmission outcome.

Diagnostic categories were not used as model predictors because diagnostic codes in routine clinical records were not considered sufficiently reliable for this purpose. In particular, diagnostic coding may be less specific during emergency psychiatric admissions and initial hospitalizations, when diagnostic certainty may be limited. Therefore, to reduce potential misclassification arising from diagnostic coding and to capture clinically relevant information documented in the admission records, the analysis focused on symptom-based clinical themes rather than diagnostic categories.

All analyses were performed in Python 3.14.4 (Python Software Foundation, Wilmington, DE, USA). Data handling used pandas 3.0.3 and NumPy 2.5.0, and source Excel files were read with openpyxl 3.1.5 and xlrd 2.0.2. Univariate tests (chi-square, Mann–Whitney U and Spearman correlation) were performed with SciPy 1.18.0, and multivariable logistic and linear regression models were fitted with statsmodels 0.14.6. Machine learning models, TF-IDF vectorization and cross-validation were implemented in scikit-learn 1.9.0. Lemma-based themes were generated using the Zeyrek morphological analyzer (version 0.1.3; O. Bulat; a Python port of Zemberek-NLP). BERTurk embeddings (dbmdz/bert-base-turkish-uncased; Bavarian State Library [MDZ], Munich, Germany; obtained from the Hugging Face Hub) were extracted on a GPU in Google Colaboratory (Google LLC, Mountain View, CA, USA) using PyTorch 2.11.0 and Transformers 5.16.1. All open-source packages were obtained from the Python Package Index (https://pypi.org). A fixed random seed (42) was used throughout.

In this study, clinical text refers to the unstructured narrative information documented in discharge summaries, particularly patients’ presenting complaints and clinical history. Twelve clinically relevant themes were identified: medication non-adherence, suicidal ideation or suicide attempt, psychotic symptoms, substance/alcohol use, family psychiatric history, previous psychiatric hospitalization, marital problems, unemployment, lack of social support, manic symptoms, physical comorbidity, and depressive symptoms. To reduce false-positive matches, a bidirectional negation detection algorithm was implemented to identify negation expressions occurring before or after keywords. During the development phase, the negation rules underwent two rounds of rule refinement based on the review of regular expression (regex) matching examples. In the first round, the negation patterns were expanded to capture inflected forms, while in the second round, three additional negation expressions were incorporated into the rules. This process was conducted as part of software development and debugging. All themes were subsequently coded as binary (present/absent) variables. Because discharge summaries contained spelling variations and clinical abbreviations, the corpus was reviewed for misspellings and out-of-vocabulary terms. Misspellings affecting predefined keywords were infrequent and were considered unlikely to influence theme extraction because each theme was defined at the patient level and was typically documented multiple times. In addition, the minimum document frequency threshold (min_df = 5) applied during TF-IDF feature generation excluded most rare terms and typographical errors. Common clinical abbreviations were incorporated into the corresponding themes, whereas institution-specific abbreviations were masked during anonymization.

Class imbalance (106 events among 622 patients; 17.0%) was addressed using class weighting and decision-threshold adjustment. No oversampling, undersampling, or synthetic minority oversampling technique (SMOTE) was applied. Class weights were defined within the modeling pipeline and, together with feature scaling, categorical encoding, and the TF-IDF vocabulary, were recalculated using only the training portion of each cross-validation fold. The data were split using stratified sampling to preserve the event rate across folds. The decision threshold was determined using the Youden J index within nested cross-validation: for each outer fold, the threshold was selected using only the training portion of that fold and then applied to the held-out portion.

Univariate analyses were performed using the chi-square test (with continuity correction for 2 × 2 tables) for categorical variables, the Mann–Whitney U test for length of hospital stay, and Spearman’s correlation analysis for age group. Variables with *p* < 0.25 in the univariable analyses were considered candidates for inclusion in the multivariable logistic regression model, in accordance with the purposeful variable selection approach described by Hosmer and Lemeshow [21]. Age group and sex were additionally included as basic sociodemographic variables, irrespective of their univariable *p*-values. In addition, variables considered clinically relevant and supported by the previous literature were included in the multivariable model regardless of their univariable statistical significance (psychotic symptoms and physical comorbidity). The final model included nine predictors and 106 readmission events, yielding an events-per-variable (EPV) ratio of 11.78. As a secondary analysis, factors associated with index hospitalization length of stay were evaluated using multivariable linear regression after logarithmic transformation of hospital stay.

Machine learning analyses compared logistic regression (structured clinical variables alone and structured clinical variables combined with TF-IDF features), Random Forest, and HistGradientBoosting models. Model performance was evaluated using repeated stratified 5-fold cross-validation with 10 repetitions. To prevent data leakage, TF-IDF vectorization was performed separately within each training fold. The optimal classification threshold for the best-performing model was determined using the Youden J index based on out-of-fold predictions. Sensitivity analyses were conducted using five alternative text representation approaches: context-restricted keyword themes, expanded keyword themes, lemma-based themes generated using the Zeyrek morphological analyzer(version 0.1.3; O. Bulat; a Python port of Zemberek-NLP); TF-IDF bag-of-words representations (raw and lemmatized text), and contextual embeddings generated using the pretrained BERTurk model. BERTurk was used solely for feature extraction and was not fine-tuned because of the limited sample size and number of outcome events.

## 3. Results

A total of 622 patients were included in the study, of whom 334 (53.7%) were female and 288 (46.3%) were male. The largest age groups were 31–40 years (*n* = 140) and 41–50 years (*n* = 131). The mean length of the index hospitalization was 14.0 ± 11.1 days, with a median of 12 days (range, 1–101 days).

The study cohort selection process is presented in Table 1.

The distribution of 1-year psychiatric readmissions according to calendar-quarter intervals is presented in Table 2.

Of the 622 patients, 106 (17.0%) had a definite readmission within 1 year, while 12 (1.9%) cases were classified as indeterminate and excluded from the primary analysis.

In the univariate analysis, previous psychiatric hospitalization, longer length of hospital stay, and manic symptoms were significantly associated with 1-year psychiatric readmission. Patients with a history of previous psychiatric hospitalization had a higher readmission rate than those without such a history (26.6% vs. 13.5%), corresponding to more than a twofold increase in the odds of readmission (OR = 2.33, *p* < 0.001). Patients who were readmitted also had a significantly longer index hospitalization (mean 16.2 vs. 13.6 days, *p* = 0.012). Similarly, patients with manic symptoms had a higher readmission rate than those without manic symptoms (27.9% vs. 15.9%; OR = 2.05, *p* = 0.029). No significant associations were observed between 1-year psychiatric readmission and depressive symptoms medication non-adherence, psychotic symptoms, physical comorbidity, suicidal ideation or suicide attempt, lack of social support, unemployment, marital problems, or substance/alcohol use (*p* > 0.05) (Table 3).

In the multivariable logistic regression analysis, previous psychiatric hospitalization was the only independent factor associated with 1-year psychiatric readmission. Patients with a history of previous psychiatric hospitalization had approximately a twofold higher likelihood of readmission within 1 year than those without such a history (adjusted OR = 2.11, 95% CI: 1.32–3.37; *p* = 0.002). No independent associations were observed between 1-year psychiatric readmission and manic symptoms, depressive symptoms, medication non-adherence, psychotic symptoms, physical comorbidity, age group, sex, or length of hospital stay (*p* > 0.05) (Table 4).

In the multivariable linear regression analysis using log-transformed length of hospital stay as the outcome, previous psychiatric hospitalization, unemployment, and depressive symptoms were independently associated with longer hospital stays. Previous psychiatric hospitalization was associated with a 50.9% increase in length of stay (*p* < 0.001), while unemployment and depressive symptoms were associated with increases of 39.9% (*p* = 0.012) and 22.2% (*p* = 0.005), respectively. Physical comorbidity, age group, and sex were not independently associated with length of hospital stay (*p* > 0.05) (Table 5).

Among the evaluated machine learning models, logistic regression using structured and theme-based features showed the best cross-validation performance, with a mean AUC of 0.602 and a mean PR-AUC of 0.253. Logistic regression with TF-IDF features showed slightly lower performance, while Random Forest and HistGradientBoosting yielded lower AUC and PR-AUC values. The BERTurk-based models showed mean AUC values of 0.550 for BERTurk embeddings and 0.548 for BERTurk embeddings combined with structured features, with mean PR-AUC values of 0.232 and 0.227, respectively. The cross-validation performance of the evaluated models is presented in Table 6.

## 4. Discussion

In this study, we aimed to explore factors associated with 1-year psychiatric readmission using clinical information extracted from discharge summaries of patients hospitalized in a psychiatric inpatient unit. In the univariate analyses, previous psychiatric hospitalization, longer index length of stay, and manic symptoms were significantly associated with 1-year readmission. However, in the multivariable analysis, only previous psychiatric hospitalization remained an independent predictor of readmission. In addition, previous psychiatric hospitalization, unemployment, and depressive symptoms were independently associated with longer hospital stays. Among the machine learning models, logistic regression using structured clinical variables and knowledge-driven theme extraction achieved the highest observed discrimination, whereas TF-IDF, Random Forest, HistGradientBoosting, and BERTurk-based text representations did not improve model performance. These findings suggest that, in the present dataset, more complex natural language processing and machine learning approaches did not outperform simpler models based on clinically meaningful and interpretable features.

The 1-year psychiatric readmission rate in the present study was 17.0%. Readmission rates among psychiatric patients are known to vary considerably depending on the diagnostic composition of the study population, follow-up duration, healthcare system organization, and the operational definition of readmission. A recent systematic review reported that 30-day unplanned psychiatric readmission rates ranged from 5% to 43% [1], whereas studies with a 1-year follow-up generally reported substantially higher rates. In a large cohort study conducted in China, 33.8% of patients were readmitted within 1 year, with urban residence, physical comorbidity, previous psychiatric hospitalization, and hospital stays longer than 60 days identified as significant predictors of readmission [22]. Similarly, the multicenter European CEPHOS-LINK study reported 1-year psychiatric readmission rates ranging from 33.2% to 47.9% across participating countries [23]. Compared with these findings, the readmission rate observed in the present study was relatively low. This discrepancy may be explained by methodological factors, including the use of a proxy measure based on calendar quarters to define the 1-year follow-up period and the single-center design, which prevented the identification of readmissions occurring at other hospitals. In addition, differences in discharge planning, access to outpatient mental health services, treatment adherence, family and social support, and socioeconomic conditions may also have contributed to the lower readmission rate observed in our cohort.

The finding that previous psychiatric hospitalization remained the only independent predictor of readmission in the multivariable analysis is consistent with the existing literature. Previous psychiatric hospitalization has been repeatedly identified as one of the strongest and most consistent predictors of psychiatric readmission across a wide range of studies [8,9,11,24]. This association may reflect underlying clinical characteristics linked to recurrent hospitalization, including a more chronic or relapsing course of illness, poorer treatment adherence, greater functional impairment, and increased need for psychosocial support.

Previous studies have shown that diagnostic categories such as schizophrenia, bipolar disorder, other psychotic disorders, and mood disorders are associated with an increased risk of psychiatric readmission [25,26,27]. In addition, psychiatric comorbidities, suicidal ideation or self-harm, a history of childhood psychological problems, and early onset of psychiatric illness have been identified as clinical factors associated with a higher likelihood of readmission [28,29,30]. In the present study, manic symptoms were significantly associated with readmission in the univariate analysis but did not remain significant after multivariable adjustment. This finding may be explained by the substantial shared variance between previous psychiatric hospitalization and symptom-related variables, particularly in chronic and recurrent disorders such as bipolar disorder where previous hospitalization likely captures much of the underlying disease burden associated with future readmission.

The absence of a significant association between suicidal ideation and 1-year psychiatric readmission in the present study is not entirely inconsistent with the existing literature. While some studies have identified suicidal ideation as an important predictor of psychiatric readmission [31], others have failed to demonstrate a significant association [28]. Moreover, the predictive value of suicidal ideation appears to be time-dependent, with a stronger association during the early post-discharge period that gradually diminishes over longer follow-up intervals [32]. Therefore, the 1-year follow-up period used in the present study may not have been sufficiently sensitive to capture the short-term predictive effect of suicidal ideation on psychiatric readmission.

Length of hospital stay is an important clinical outcome in the evaluation of psychiatric inpatient care. In addition to increasing healthcare resource utilization and treatment costs, prolonged hospitalization may adversely affect patients’ social and occupational functioning, community reintegration, and ability to live independently [33]. Therefore, identifying factors associated with length of hospital stay is essential for optimizing treatment planning, improving discharge management, and promoting more efficient use of healthcare resources.

In the present study, previous psychiatric hospitalization, unemployment, and depressive symptoms were independently associated with longer hospital stays. Previous studies have likewise demonstrated that a wide range of clinical and healthcare system-related factors, including age, sex, psychiatric diagnosis, history of previous hospitalization, level of functioning, illness severity, hospital characteristics, and health insurance status, may influence length of hospital stay [34,35]. However, determinants of hospital stay have been shown to vary across countries and healthcare systems [15]. Therefore, the findings of the present study should be interpreted in the context of data obtained from a single university hospital in Turkey and may not be directly generalizable to other healthcare settings.

Although unemployment was not associated with 1-year psychiatric readmission, it was independently associated with longer hospital stays. This finding is consistent with previous evidence suggesting that unemployment may be associated with prolonged psychiatric hospitalization [36,37]. Tulloch et al. reported that unemployment and other social circumstances may be related to length of stay partly through their association with illness severity and functional impairment, as these factors may influence clinicians’ perceptions of the need for continued hospitalization [13]. In this context, unemployment may reflect greater functional or social difficulties that complicate discharge planning and social reintegration, thereby contributing to longer hospitalization. On the other hand, previous studies have also identified unemployment as a predictor of psychiatric readmission [1,10]. The discrepancy between these findings and our results may partly reflect differences in the demographic and socioeconomic composition of the study populations. In our cohort, the distribution of demographic and socioeconomic characteristics was relatively limited, and several potentially relevant accompanying sociodemographic factors, such as educational attainment and detailed socioeconomic status, were not systematically documented. These factors may have influenced the observed association between unemployment and the different outcomes and should be considered in future studies.

Similarly, depressive symptoms were not independently associated with one-year psychiatric readmission but were associated with longer hospital stay. This finding is consistent with previous research showing that greater depressive symptom severity is associated with prolonged psychiatric hospitalization. In one study of inpatients with psychotic, depressive, and bipolar disorders, depressive symptoms assessed using the Brief Psychiatric Rating Scale were associated with longer hospital stays across all three diagnostic groups [38]. In our cohort, depressive symptoms were the most frequently identified symptom theme and may therefore represent a transdiagnostic dimension of overall psychopathology and clinical burden rather than a diagnosis-specific characteristic. Because our analysis focused on symptom-based themes rather than diagnostic categories, we could not determine whether the association between depressive symptoms and length of stay differed across specific psychiatric diagnoses.

Early identification of factors associated with prolonged hospital stay may not only facilitate more efficient use of healthcare resources but also enable the timely recognition of high-risk patients and the development of individualized treatment and discharge plans. In particular, readily assessable clinical characteristics such as previous psychiatric hospitalization, unemployment, and depressive symptoms may help clinicians better anticipate patients’ care needs, implement multidisciplinary interventions in a timely manner, and reduce unnecessarily prolonged hospitalizations.

This study has several strengths. First, it was conducted on a relatively large clinical cohort of 622 psychiatric inpatients, enhancing the reliability of the findings. Second, in addition to structured demographic and clinical variables, the study systematically incorporated information extracted from routinely documented free-text discharge summaries using a knowledge-driven, theme-based coding approach. Furthermore, the direct comparison of conventional statistical analyses with multiple machine learning approaches within the same dataset allowed an evaluation of the relative performance of clinically interpretable models and more complex natural language processing methods. Finally, by examining both psychiatric readmission and length of hospital stay, the study provides a more comprehensive assessment of clinically relevant inpatient outcomes.

Several limitations should also be acknowledged. First, because admission and discharge dates were anonymized at the calendar-quarter level, the primary outcome could only be approximated rather than determined using exact dates. Second, text-based variables were extracted using a keyword- and regular expression-based approach, which cannot fully capture the complexity of natural language. Although the negation detection rules underwent two rounds of refinement during software development, clinical validation of the extraction algorithm was not performed. Therefore, some complex linguistic structures, such as double negation, may not have been fully captured, potentially resulting in occasional misclassification of theme presence. In addition, several established predictors reported in the literature, including insight, medication adherence, and ICD-10 diagnostic codes, could not be evaluated because they were not systematically documented in the retrospective clinical records. The heterogeneity of psychiatric diagnoses within the study cohort was therefore not explicitly modeled, which may have limited the identification of diagnosis-specific predictors of readmission and contributed to residual clinical heterogeneity in the predictive models.

Moreover, the predictive performance of the developed models was modest (AUC: 0.52–0.62), indicating that the findings should be considered exploratory and hypothesis-generating. External validation using an independent cohort from another hospital or time period was not performed. The single-center design also limits the generalizability of the findings. In addition, one-year psychiatric readmission was modeled as a single binary outcome across a diagnostically and demographically heterogeneous inpatient population. Readmission may reflect different clinical processes across diagnostic and patient subgroups, which may have limited the ability of a single undifferentiated outcome to be discriminated using common clinical features and may have imposed an inherent ceiling on model discrimination. This issue is distinct from limitations in text extraction and measurement and suggests that future studies should consider not only improved text-mining approaches but also stratified modeling based on diagnostic and clinically meaningful patient subgroups.

## 5. Conclusions

In conclusion, this study demonstrated that previous psychiatric hospitalization was the only independent factor associated with 1-year psychiatric readmission among psychiatric inpatients, while previous psychiatric hospitalization, unemployment, and depressive symptoms were independently associated with longer hospital stays. Furthermore, analysis of unstructured clinical information extracted from discharge summaries using machine learning methods did not provide additional predictive value beyond models based on structured clinical variables in the present dataset. These findings suggest that routinely available clinical information may provide a basis for further investigation of psychiatric readmission risk. Future studies using larger multicenter datasets, standardized clinical documentation, and more advanced natural language processing techniques may facilitate the development of more accurate and generalizable models for predicting psychiatric readmission.

## Figures and Tables

**Table 1:** Flowchart of Study Cohort Selection.

Stage	*n*
Total psychiatric hospitalization records	1064
Unique patients	751
Patients excluded (outside the index period, 2020–2024)	129
Patients included in the study (study cohort)	622
Patients readmitted within 1 year	106
Patients not readmitted within 1 year	516
1-year psychiatric readmission rate	17.0%

**Table 2:** Calendar-quarter classification of 1-year readmission.

Quarter Interval	*n*	Classification
Same quarter	38	≤365 days
+1 quarter	37	≤365 days
+2 quarter	19	≤365 days
+3 quarter	12	≤365 days
+4 quarter	12	Indeterminate (Not readmitted)

**Table 3:** Association Between Clinical Characteristics and 1-Year Psychiatric Readmission.

Variable	Positive Group (*n*)	Readmission Rate (Positive vs. Negative Group)	Crude OR	*p*-Value
Previous psychiatric hospitalization	169	26.6%/13.5%	2.33	**0.0002**
Length of hospital stay (days)	–	Mean: 16.2/13.6	–	**0.012**
Manic symptoms	61	27.9%/15.9%	2.05	**0.029**
Depressive symptoms	298	20.1%/14.2%	1.52	0.063
Medication non-adherence	45	24.4%/16.5%	1.64	0.244
Psychotic symptoms	132	13.6%/18.0%	0.72	0.297
Physical comorbidity	74	12.2%/17.7%	0.64	0.306
Suicidal ideation/attempt	186	19.4%/16.1%	1.25	0.376
Lack of social support	11	27.3%/16.9%	1.85	0.613
Unemployment	47	17.0%/17.0%	1.00	1.000
Marital problems	80	17.5%/17.0%	1.04	0.874
Substance/alcohol use	45	17.8%/17.0%	1.06	0.839

*Note*. Bold values indicate statistically significant differences between models (adjusted *p* < 0.05).

**Table 4:** Multivariable Logistic Regression Analysis of Clinical Factors Associated with 1-Year Psychiatric Readmission.

Variable	Adjusted OR	95% CI	*p*-Value
Previous psychiatric hospitalization	2.11	1.32–3.37	0.002
Manic symptoms	1.61	0.85–3.05	0.148
Depressive symptoms	1.38	0.89–2.15	0.152
Medication non-adherence	1.22	0.57–2.59	0.610
Psychotic symptoms	0.66	0.37–1.16	0.148
Physical comorbidity	0.64	0.30–1.36	0.243
Age group (ordinal)	0.98	0.85–1.12	0.745
Female sex	1.09	0.70–1.69	0.713
Length of hospital stay (days)	1.01	0.99–1.03	0.191

**Table 5:** Multivariable Analysis of Length of Hospital Stay (Log-Transformed OLS Regression, *n* = 622).

Variable	Effect on Length of Hospital Stay (%)	*p*-Value
Previous psychiatric hospitalization	+50.9	<0.001
Unemployment	+39.9	0.012
Depressive symptoms	+22.2	0.005
Physical comorbidity	+12.9	0.273
Age (ordinal group)	+3.3	0.131
Female sex	+8.0	0.283

**Table 6:** Cross-Validation Performance and Pairwise Comparisons of Machine Learning Models.

Model	Mean AUC	SD	Mean PR-AUC
Logistic Regression (Structured + Theme Features)	0.602	0.067	0.253
Logistic Regression (+TF-IDF)	0.594	0.068	0.236
Random Forest	0.553	0.065	0.226
HistGradientBoosting	0.519	0.069	0.207
Logistic Regression (BERTurk embeddings)	0.550	0.064	0.232
Logistic Regression (BERTurk embeddings + structured)	0.548	0.064	0.227
**Comparison**	**AUC Difference**	**Adjusted 95% CI**	**Adjusted *p*-Value**
Theme-based Logistic Regression vs. Logistic Regression (+TF-IDF)	0.008	−0.027 to 0.042	0.664
Theme-based Logistic Regression vs. Random Forest	0.048	−0.028 to 0.124	0.207
Theme-based Logistic Regression vs. HistGradientBoosting	0.082	0.004 to 0.160	**0.039**
Theme-based Logistic Regression vs. Logistic Regression (BERTurk embeddings)	0.051	−0.033 to 0.135	**0.225**
Theme-based Logistic Regression vs. Logistic Regression (BERTurk embeddings + structured)	0.053	−0.029 to 0.135	**0.200**

*Note.* Bold values indicate statistically significant differences between models (adjusted *p* < 0.05).

## Data Availability

The data presented in this study are available on request from the corresponding author. The data are not publicly available due to privacy and ethical restrictions.
